# Risk profiling and efficacy of albendazole against the hookworms *Necator americanus* and *Ancylostoma ceylanicum* in Cambodia to support control programs in Southeast Asia and the Western Pacific

**DOI:** 10.1016/j.lanwpc.2021.100258

**Published:** 2021-08-26

**Authors:** Vito Colella, Virak Khieu, Andrew Worsley, Dammika Senevirathna, Sinuon Muth, Rekol Huy, Peter Odermatt, Rebecca J. Traub

**Affiliations:** 1 Faculty of Veterinary and Agricultural Sciences, The University of Melbourne, Parkville, Australia; 2 National Centre for Parasitology, Entomology and Malaria Control, Ministry of Health, Phnom Penh, Cambodia; 3 Datamahi, Melbourne, Australia; 4 Swiss Tropical and Public Health Institute, Basel, Switzerland; 5 University of Basel, Basel, Switzerland

**Keywords:** Soil transmitted helminths, *Ancylostoma ceylanicum*, Zoonosis, Control programs, Albendazole

## Abstract

**Background:** Hookworm disease is endemic throughout many parts of the Asia Pacific, despite targeted control programs of at-risk populations. The success of these programs has been hindered by the limited efficacy of widely-used mebendazole, rapid re-infection rates linked to persistent reservoirs of untreated people and dogs, and the low sensitivity of conventional coprodiagnostic techniques employed.

**Methods:** Here, we used standard faecal flotation (SFF) and a multiplex qPCR (mqPCR) assay to calculate and compare species-specific cure and egg reduction rates of single dose albendazole (400 mg) against hookworm infections at community level. Data from a cross-sectional survey in 1,232 people from Cambodia were used to inform a generalised linear mixed model to identify risk factors linked to hookworm infection(s) at baseline. Furthermore, we calculated risk factors associated to the probability of being cured after albendazole administration.

**Findings:** Overall, 13·5% of all 1,232 people tested by SFF were positive for hookworm infection(s). Most (80·1%) infected people were >12 years of age, hence above the age targeted by the WHO control program. We estimate that as age increases, the odds of being infected increases at a faster rate for females than for males. We revealed a substantial difference in cure rate of hookworm infection(s) following albendazole treatment using the SFF (81·5%) and mqPCR (46·4%) assays, and provide the first data on the efficacy of this drug against the zoonotic hookworm *Ancylostoma ceylanicum*. We estimated that as age increases by one year, the odds of being cured decreases by 0·4%–3·7%. Similarly, the odds of being cured for people who boiled drinking water was estimated to be between 1·02 and 6·82.

**Interpretation:** These findings show that the adoption of refined diagnostic techniques is central to monitoring hookworm infection(s) and the success of control strategies, which can ultimately aid in reducing associated morbidity in human populations. The approach taken is likely to be directly applicable to other parts of Southeast Asia and the Western Pacific, where specific epidemiological conditions might hamper the success of targeted treatment programs.

**Funding:** Faculty of Veterinary and Agricultural Sciences Strategic Research Funds, The University of Melbourne.

**RESEARCH IN CONTEXT**

**Evidence before this study**

Infections with soil-transmitted helminths (STHs) affect 2 billion people worldwide resulting in a disease burden of ∼4 million DALYs. Hookworm disease causes the highest public health burden among STHs, being the most prevalent of the neglected tropical diseases. The disease affects nearly half a billion people living in low socioeconomic settings, with children and women of child-bearing age being disproportionately impacted. Control relies heavily on the reduction of morbidity through periodic treatment with albendazole or mebendazole of at-risk populations. In Southeast Asia alone, 297 million school-age children were treated, representing 90% coverage of the total population. However, despite decades of mass drug administration hookworm in children remains endemic in many parts of the Asia Pacific.

In this region the presence of untreated canids serving as reservoirs of *Ancylostoma ceylanicum* hookworm increases the rapid re-infection of humans. Unlike the human hookworms *Necator americanus* and *Ancylostoma duodenale, A. ceylanicum* is zoonotic and is the second most common hookworm in many regions in Southeast Asia and Western Pacific infecting ∼100 million people.

Thus, the success of control programs is hindered by low efficacy of mebendazole, the rapid re-infection rates from persistent reservoirs of untreated individuals and free-roaming dogs, and the low sensitivity of microscopy-based diagnostic techniques (unable to distinguish between human and zoonotic hookworms).

Given the well-known poor sensitivity of microscopy and the large differences in the epidemiology of human and zoonotic hookworm species, the identification of these parasites at species level through molecular methods should be the mainstay of risk factor and efficacy analyses when evaluating impact of current control strategies.

We performed a community-wide cross-sectional survey to assess prevalence of hookworm infections using conventional faecal flotation, and a multiplex quantitative PCR (mqPCR) assay for the identification and quantification of hookworm at species level. We calculated the cure and egg reduction rates of single dose albendazole against hookworm infections using traditional techniques and the mqPCR to assess species-specific efficacy. Generalized linear mixed models were used to identify risk factors related to hookworm infections and to the probability of being cured after albendazole administration.

**Added value of this study**

We provide the first efficacy data of albendazole against the zoonotic *A. ceylanicum* in people living in the Asia Pacific with the aid of refined diagnostic techniques. We report a large difference in the cure rates of albendazole against hookworm infections detected by conventional (81·5%) and molecular (46·4%) methods. The low sensitivity of conventional diagnostic methods currently adopted on a global scale, incorrectly classifies the majority of individuals as cured, thus overestimating efficacy of anthelminthic drugs used during mass deworming. Additionally, we provide the first data on egg reduction rates of hookworm infections at species level after drug administration.

In these Cambodian communities, 80·1% of the infected population was above 12 years of age targeted by the WHO control strategies. We estimate that as age increases by one year the odds of being infected increase while the odds of being cured decrease, leaving a large population of untreated individuals susceptible to the infection.

**Implications of all the available evidence**

This study provide evidence that the application of appropriate diagnostic techniques and analytical approaches to monitor hookworms in entire communities is fundamental to support the development of refined strategies to reduce morbidity of hookworm infections especially in Southeast Asia and the Western Pacific where specific epidemiological conditions may hamper the success of targeted deworming. Data generated from risk profiling and cure rates of albendazole against hookworm at species level in individuals from villages in Cambodia provide policy makers with substantial information to support re-evaluation of diagnostics methods and control strategies for hookworms in Southeast Asia and the Western Pacific.

Alt-text: Unlabelled box

## Introduction

Infections with soil-transmitted helminths (STHs; *Ascaris lumbricoides, Trichuris trichiura* and/or hookworms) affect ∼ 2 billion people worldwide, resulting in a disease burden of ∼ 4 million disability adjusted life years (DALYs).^1^^,^^2^ Hookworm disease alone affects nearly half a billion people living in underprivileged communities, and is recognised as one of the most prevalent neglected tropical diseases (NTDs).^1^ The disease is caused by blood-feeding roundworms (nematodes) of the small intestine, and contributes to iron deficiency anaemia and malnutrition, with children and women of child-bearing age (WCBA) being disproportionately impacted.^2^ As no vaccine is presently available, control relies heavily on the reduction of morbidity through periodic treatment with single oral doses of albendazole or mebendazole, targeting at-risk populations. ^3^ Treatment is mainly focused on school-age children (SAC), because they are at more risk of morbidity (e.g. anaemia, impaired physical and cognitive development) than older people, and because anthelmintic treatment is facilitated by the use of school-based infrastructure and personnel. ^4^^,^^5^ Although WCBA would benefit substantially from preventative chemotherapy (PC), such an approach has not yet been widely implemented.

In 2017, 596 million SAC required PC globally, with > 50% of these children living in the most disadvantaged regions of Southeast Asia and the Western Pacific.^6^ In Southeast Asia alone, 297 million SAC were treated in areas requiring PC, representing 90% coverage of the total population. ^6^ However, in spite of sustained efforts, hookworm infection in children remains endemic in many parts of the Asia Pacific,[6], [7], [8] owing, in part, to poor anthelmintic efficacy of widely-used mebendazole (single oral dose),^9^ and rapid re-infection rates linked to persistent reservoirs of untreated people.

Recent evidence challenges the long-term impact of current, age-targeted control strategies on hookworm morbidity, because community-wide transmission is bolstered by large populations of untreated adults, and adoption of mass-drug administration has been recommended to support sustained reduction of morbidity.[10], [11], [12], [13] This scenario is complicated in Southeast Asia and the Western Pacific by the presence of canids serving as reservoirs of hookworm infections. ^13^ These animals live in close proximity to human dwellings and contribute to environmental contamination with larvae of *Ancylostoma ceylanicum.*[14], [15], [16] Unlike the hookworms *Necator americanus* and *Ancylostoma duodenale, A. ceylanicum* is zoonotic and is the second most common hookworm infecting humans in many regions in Southeast Asia and the Western Pacific, including the Solomon Islands^17^, Lao People's Democratic Republic (Lao PDR)^18^, Malaysia^19^, Cambodia^20^, Myanmar^21^^,^^22^ and also The Philippines.^23^

To date, single dose regimens of albendazole (400 mg) remains the most efficacious and widely–used anthelmintic against hookworm, with an average cure rate (CR) of 79·5% and egg reduction rate (ERR) of 89·6%.^24^ Nonetheless, efficacy studies have considered the three main hookworm species collectively, owing to the limitations of conventional diagnostic techniques (i.e. microscopy) being unable to distinguish among these species.^15^ Given the poor diagnostic sensitivity and specificity of conventional coproscopic methods (e.g., Kato Katz and faecal floatation)^25^ and the marked differences in the biology and epidemiology of human and zoonotic hookworm species, the identification of these parasites using molecular methods should be the mainstay of risk factor and efficacy analyses when evaluating the impact of current control strategies.

In this study, we performed a community-wide cross-sectional survey to assess the overall prevalence of hookworm infections using standard faecal flotation (SFF)^26^ and a multiplex qPCR (mqPCR) assay to differentiate *N. americanus, A. ceylanicum* and *A. duodenale* infections. We calculated the CR of single dose albendazole (400 mg) against hookworm infections using traditional techniques and the mqPCR to assess species-specific efficacy one-week after the treatment. Generalised linear mixed models were used to identify risk factors linked to hookworm infection(s) and to the probability of being cured after albendazole administration.

The data generated from risk profiling and cure rates of albendazole against hookworm at species level in individual people from villages in Cambodia provide policy makers with substantial information to support refined control strategies in Southeast Asia and the Western Pacific.

## Methods

### Ethical considerations

The study was approved by the National Ethics Committee for Health Research (NECHR; number 269NECHR, dated 27 June 2016), Ministry of Health, Cambodia and The University of Melbourne Human Research Ethics Committee (ID: 1647208). All relevant authorities and all study participants were informed about the purpose and procedures of the study. Written informed consent was obtained from the participants aged 18 years and older and from parents or the legal guardian of children aged under 18 years, after having received their assent to participate in the research. All individuals infected with hookworms were treated with a single dose of Albendazole 400 mg (Alzental, Shing Poong Pharm. Co. Ltd, Korea) except pregnant women in the first trimester according to the Cambodian national helminth control program.

### Study setting and population

The study was carried out in ten purposively selected rural villages in Rovieng district (13°30′N 104°50′E), Preah Vihear province, northeastern Cambodia, namely Chambak Pa'em, Chamlong, Kampot, Rovieng Tboung, Sangker Roung, Pal Hal, Anglong Svay, Chamlong, Bos Pey and Sra'er Thom. The cross-sectional survey was conducted during the dry season from June to July 2016. Rice and subsistence farming are the main economic activities in the villages. Pigs, poultry, and cattle are the most common domestic animals. Dogs and cats are common free-roaming animals in the villages sharing the same environment as that of people.

With a simple random sampling, a sample size of 1,192 people was required to obtain a 95% CI of ±2·5% for a prevalence estimate of 26·5% hookworm-infected people in Cambodia. ^20^ Sample size for cure rate (CR) was based on a previous hookworm efficacy study in neighbouring Lao PDR that indicated a single oral dose of albendazole produced a CR of ∼36%. ^27^ A sample size of 84 individuals infected with hookworms was calculated based on a conservative CR estimate of 30% for single dose albendazole against hookworms with 95% CI.

**Figure 1:**
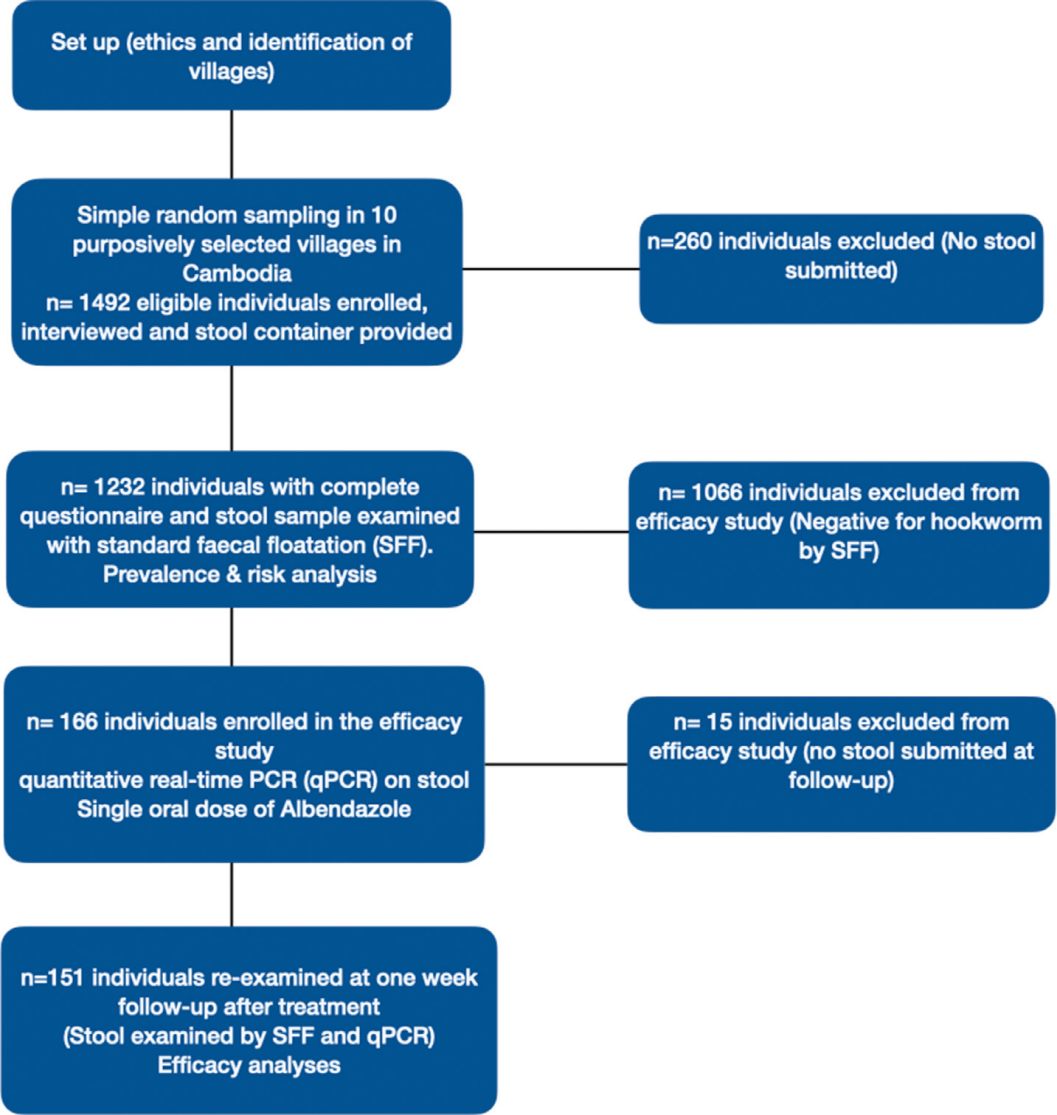
Flow diagram of the study design.

The head of the village was informed about the study procures and dates and provided information to the villagers. On the survey day, the villagers were met at central location (pagoda) and enrolled. All individual presenting on the survey day older than 6 years were eligible to participate. After written informed consent was obtained, field workers interviewed the enrolled participants by using a pre-tested questionnaire. For participants younger than 18 years the consent was obtained from guardian or parents. Parents answered the questionnaire for children aging less than 12 years. A pre-labelled (ID code, name, age, sex, date) stool container was provided to each study participants in the afternoon-evening, together with instructions on how collect faecal samples. On the morning after, the stool containers were collected and transported to the local laboratory at the health centre in Rovieng and analysed by SFF. A flow diagram of the study design is illustrated in Figure 1.

### Treatment of hookworms‐infected patients and follow up

Individuals that were positive for hookworm by SFF were administered a single oral dose of albendazole 400 mg (Alzentel, **Shin Poong Pharm. Co. Ltd., Korea**) by directed observation of trained field workers from the National Centre for Parasitology, Entomology and Malaria Control (CNM) Ministry of Health, Cambodia and asked to submit a second stool sample after the first week. The expiry date of the albendazole was 14 November 2018 (Manufactured batch: ALZET3003). The stool sample was examined by SFF and mqPCR at baseline and follow-up (see below).

The WHO advises follow-up sampling of anthelmintic efficacy studies be performed no later than 14-21 days to ‘avoid the risk that eggs identified in a specimen are from parasites that infected the individual after drug administration’.^28^ Accordingly, we performed a follow-up after the first week given no significant difference exists in hookworm egg output in human stool samples collected from the first to the third^29^ or to the seventh week^30^ following drug administration.

### Microscopic and molecular detection of hookworms

Within 60 min of collection, the stool samples were transported to the mobile field laboratory at the Rovieng Health Centre at room temperature. Upon arrival, the samples were refrigerated at 4°C for a maximum of 4 hours before experienced laboratory technicians examined the specimens. The stool samples were screened and enumerated for hookworm eggs expressed in egg per gram faeces (EPG) using SFF.^26^ An aliquot of stool (∼ 2 g of faeces) from individuals positive for hookworms at SFF was preserved in 5% potassium dichromate in 15 ml centrifuge tubes. These samples were refrigerated at 4°C and exported in this condition to the University of Melbourne, Australia for molecular analysis. Individuals found microscopy-positive at follow-up were re-treated with a single oral dose of 400 mg albendazole. The same laboratory procedures were performed at follow-up as per baseline.

At the University of Melbourne, 250 mg faeces were washed in 1% PBS and subjected to DNA extraction using the Faecal Isolate II DNA extraction kit (Bioline, London, United Kingdom) according to manufacturer instructions. Faecal DNA was screened for hookworm species using a mqPCR reaction, each reaction run in duplicate using primers/probes NecF (CTGTTTGTCGAACGGTACTTGC) and Nec R (ATAACAGCGTGCACATGTTGC) and Nec Probe (/5Cy5/CTG+TA+CTA+CG+CAT+TGTATAC/3IAbRQSp/) for *N. americanus* and Anc F (CGGGAAGGTTGGGAGTATC) and Anc R (CGAACTTCGCACAGCAATC) and A.cey Probe (/56-FAM/CCGTTC+CTGGGTGGC/3IABkFQ/) and A.duo Probe (/5HEX/TCGTTAC+T+GGGTGACGG/3IABkFQ/) for *Ancylostoma* spp. Primers EHV F (GATGACACTAGCGACTTCGA) and EHV R (CAGGGCAGAAACCATAGACA) and probe EHV Probe EHV (5′ /ROX/TTTCGCGTGCCTCCTCCAG/3IAbRQSp/-3) were used to amplify Equine herpesvirus type 4.

Genomic DNA as synthetic block gene fragments (IDT® Technologies, Skokie, Illinois, USA) containing the DNA target sequences for *A. ceylanicum, N. americanus* and *A. duodenale* were used as positive controls in each run. Equine herpesvirus type 4 (EHV4) was used as an internal amplification control and a human DNA qPCR using primers VERTF 5’ CGAACGTCTGCCCTATCAAC3’, VERTR 5’ CGTTTCTCAGGCTCCCTCT3’ and HumanProbe /56-FAM/CCCGATGGT/ZEN/GCAGCCGCTATTAAA/3IABkFQ/ (IDT® Technologies, Skokie, Illinois, USA) served as DNA extraction control. For a stool sample to be considered positive for infection, a limit of detection of cut-off was set to cycle threshold (Ct) 33.^31^ An assay was deemed a failure when the Ct value of the EHV4 PCR in the sample was greater by two or more cycles compared with the negative control EHV4 Ct value. Magnetic Induction Cycler (Bio Molecular Systems, Sydney, Australia) was used for the amplification, detection, and data analysis. Methods outlined in ^31^ were used to convert Ct to absolute egg counts for *A. duodenale* and *N. americanus*, as follows. EPG of faeces were calculated from Ct-values using the formulas log_10_epg = -0·1844x Ct + 6·1145 for *N. americanus* and log_10_epg = -0·1731x Ct + 6·4174 for *A. ceylanicum*.

### Statistical analyses

Interview and laboratory data were double-entered in EpiData version 3.1 (EpiData Association; Odense, Denmark) and validated. Only participants with a complete record (stool samples at baseline and follow-up examined with both diagnostic methods and complete questionnaire information) were retained for anthelmintic efficacy analysis.

The CR against hookworms was calculated as percentage of hookworm positive individuals who became egg-negative after treatment calculated by SFF and mqPCR (Number of individuals positive pre-treatment and negative post-treatment / Number of individuals positive pre-treatment × 100). The prevalence of hookworm infection was calculated for SFF method results only.

95% binomial exact confidence intervals were calculated for both prevalence and CR. Age groups (6–11 years; 12–17 years; 18–64 years; and > 65 years) and sex were examined separately for potential associations with the probability of being cured, using the Pearson's Chi-squared test, or the Fisher's Exact Test if cell counts were low.

Egg Reduction Rate (ERR) was measured as percentage of mean reduction (both arithmetic AM and geometric mean GM) of eggs at follow-up compared to baseline by SFF (total hookworm) and mqPCR (species-specific hookworm). Confidence intervals AM and GM ERR were calculated using a bootstrap re-sampling method with 10,000 replicates.

The impact of baseline EPG on AM and GM ERR was assessed using a multivariable linear regression model, adjusted for age, sex and community membership.

Age-prevalence at baseline and cure rate-age smoothed curves were generated using LOESS Curve Fitting (Local Polynomial Regression) and a generalized linear mixed model (logit link function) with a random group intercept for communities was used to find and estimate the effects of potential risk factors on infection and on the odds of being cured from each hookworm species using R version 3.6.1, lme4 version 1.1-23. For both the multivariable linear and generalized linear mixed methods a strategy of backward stepwise variable selection was used to arrive at the final model. This starts with a full variable set then the least significant variable is removed at each step with the objective being to arrive at a model with all variables statistically significant, this being deemed adequate for the inference of effects.

This study is reported as per Strengthening the Reporting of Observational Studies in Epidemiology (STROBE) guidelines.^32^

### Role of the funding source

This study was funded by the Faculty of Veterinary and Agricultural Sciences Strategic Research Funds, The University of Melbourne. The funders had no role in study design, data collection, data analysis, interpretation, and writing of the report.

## Results

For the 10 villages studied, 1,492 randomly selected individuals were present and eligible for study participation, 1,232 (84·3%) of whom agreed to participate and submitted a stool sample (Figure 1). Participants ranged in age from 6 to 90 years, with a median of 27 years and interquartile range of 33. Of the study participants, 57·4% were females and 42·6% were males. Characteristics of the 1,232 study participants are summarised in Table 1.

**Table 1:** Characteristics of 1,232 study participants from villages of Rovieng district, Preah Vihear province, northeastern Cambodia.

Variable	Category	n (%)
Age		
	6-11	299 (24·3)
	12-17	207 (16·8)
	18-64	619 (50·2)
	65+	107 (8·7)
Sex		
	Male	525 (42·6)
	Female	707 (57·4)
Occupation		
	Farmer	642 (52·1)
	Student	474 (38·5)
	Teacher	16 (1·3)
	Other	100 (8·1)
Education		
	None	112 (9·1)
	Primary	868 (70·5)
	Secondary	171 (13·9)
	High School	79 (6·4)
	University	2 (0·2)
Dogs or cats in the house		
	Yes	1053 (85·5)
	No	179 (14·5)
Frequency of visiting rice fields		
	Never	229 (18·6)
	Rarely	23 (1·9)
	Sometimes	250 (20·3)
	Often	730 (59·3)
Dog to the rice field		
	Never	614 (49·8)
	Rarely	24 (1·9)
	Sometimes	388 (31·5)
	Often	206 (16·7)
Main water source		
	Lake	27 (2·2)
	Well	1205 (97·8)
Main source of drinking water		
	Lake	637 (51·7)
	Well	509 (41·3)
	Pure	83 (6·7)
	No answer given	3 (0·2)
Boiling water during the wet/rainy season		
	No	883 (71·7)
	Yes	349 (28·3)
Drinking water in the dry season		
	Well	1137 (92·3)
	Pure	81 (6·6)
	Lake	13 (1·1)
	No answer given	1 (0·1)
Boiling water during the dry season		
	No	838 (68·0)
	Yes	393 (31·9)
	No answer given	1 (0·1)
Frequency of using shoes		
	Sometimes	13 (1·1)
	Often	1204 (97·7)
	Always	14 (1·1)
	No answer given	1 (0·1)
Treated with any anthelminthic drugs in the last two months		
	No	737 (59·8)
	Yes	495 (40·2)
Toilet at home		
	No	439 (35·6)
	Yes	793 (64·4)
Place used to defecate		
	Toilet	803 (65·2)
	Rice field	78 (6·3)
	Forest	169 (13·7)
	Other	182 (14·8)

### Baseline prevalence and predictors for hookworm infections

In total, 166 individuals were SFF-positive for hookworm eggs with an overall prevalence of 13·5% (95% CI: 11·6-15·5). The proportion of people diagnosed with hookworm infection per age class and sex are summarised in Table 2; 80·1% (95% CI: 73·2-85·9) of the infected population was >12 years of age. No statistically significant difference was found between infection at the baseline and age classes, and males were more likely to be diagnosed with hookworm infection than females (OR 2·0, 95 % CI: 1·5-2·8; P<0·0001).

**Table 2:** Number and percentages of people diagnosed with hookworm infection by microscopy in villages of Rovieng district, Preah Vihear province, northeastern Cambodia stratified per age classes and sex.

Variable	Category	n (%)
Age		
	6-11	33 (11·0)
	12-17	27 (13·0)
	18-64	86 (13·9)
	65+	20 (18·7)
Sex		
	Male	*96 (18·3)
	Female	70 (9·9)

*p<0·0001

Using a generalised linear mixed model, we found evidence of an interaction between age and sex, and weak evidence of an association between the fixed effect of boiling drinking water during the wet season, on baseline infection, holding all other variables constant (Table 3).

**Table 3:** Coefficient estimates of community-adjusted effects for hookworm infection at baseline in 1,232 study participants and cure rates in infected people from villages of Rovieng district, northeastern Cambodia.

Baseline	Coefficient Estimate (95% CI)	*p* value
Intercept	-1·777 (-2·315, -1·304)	<0·0001
Age (years)	0·006 (-0·006, 0·017)	0·3195
Sex (Female)	-1·368 (-2·074, -0·691)	<0·0001
Boiling water during wet season	-0·426 (-0·902, 0·031)	0·0713
Age (years) x Sex (Female)	0·017 (0·001, 0·034)	0·0394
Cure rates		
Intercept	0·265 (-0·518, 1·034)	0·484
Age (years)	-0·020 (-0·038, -0·004)	0·020
Boiling water during wet season	0·949 (0·026, 1·919)	0·047

We estimate that as age increases, the odds of being infected increases at a faster rate for females than for males, holding sex and boiling water in the wet season constant, while adjusting for the community effect. Boiling water in the wet season was significant at the 10% level, with the odds ratio of infection being between 0·44 and 0·96, holding sex and age constant, while adjusting for the community effect (90% CI).

### Assessment of CRs and ERRs as calculated by SFF and mqPCR

In total, 151 individuals provided sufficient volumes of both pre- and post-treatment samples subjected to both SFF and mqPCR-based screening, which were included in the efficacy analyses. At baseline, 131 and 8 individuals were infected with single infections of *N. americanus* (86·8%) and *A. ceylanicum* (5·3%), respectively, and 12 individuals had infections with multiple species, 11 with *N. americanus* and *A. ceylanicum* (7·3%) and a single individual with *N. americanus* and *A. duodenale* (0·6%)*.*

**Figure 2:**
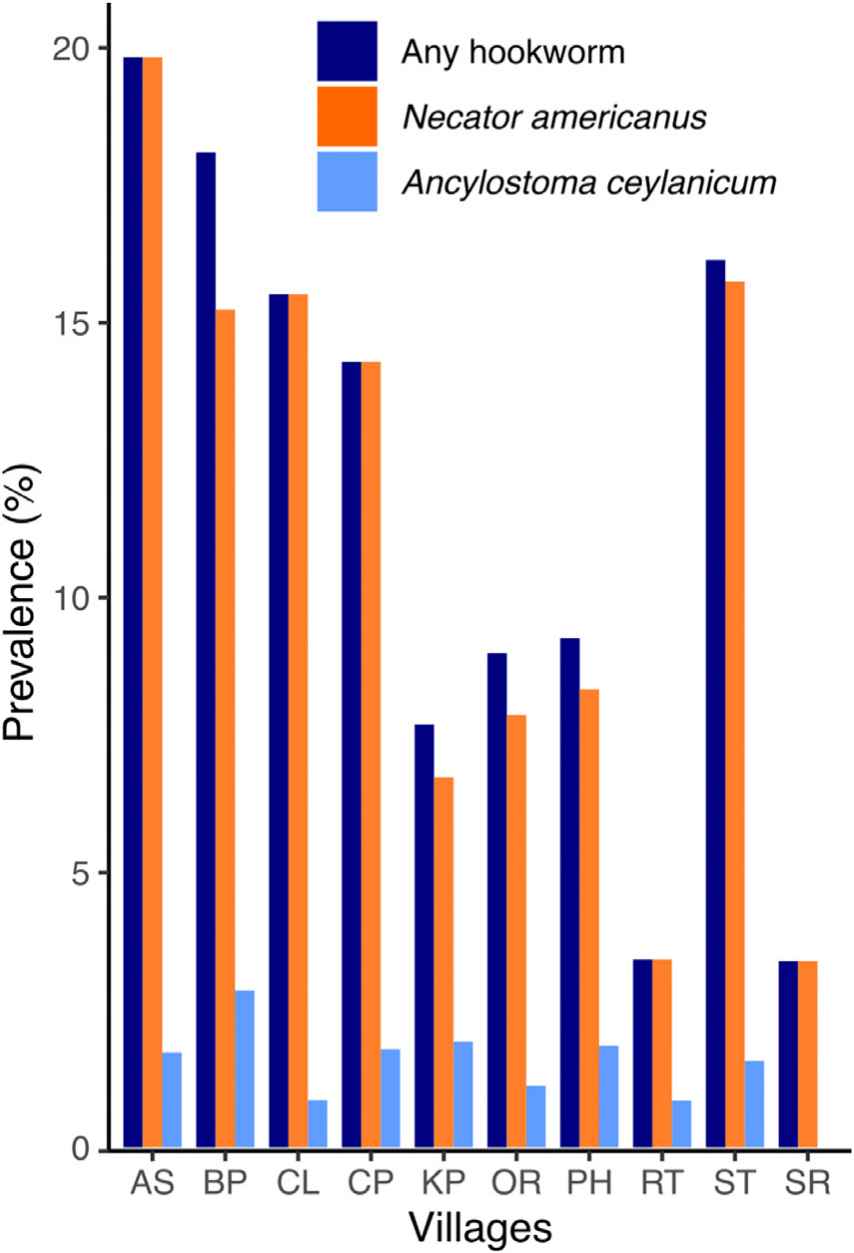
Community-based prevalence to any hookworm, *Ancylostoma ceylanicum*, and *Necator americanus* as calculated by mqPCR in Anglong Svay (AS), Chamlong, Bos Pey (BP), Chamlong (CL), Chambak Pa'em (CP), Kampot (KP), Or (OR), Pal Hal (PH), Rovieng Tboung (RT), Sra'er Thom (ST), and Sangker Roung (SR) villages.

**Figure 3:**
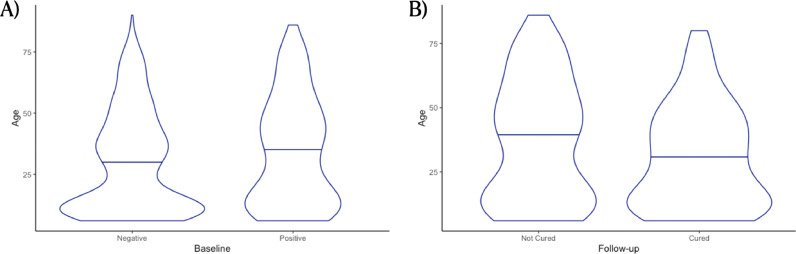
Age distribution of individuals based on the infection status at baseline (A) and on the outcome of being cured (B) from any hookworm infection.

Overall, the community-level prevalence for any hookworm and for *N. americanus* alone, as calculated by mqPCR on SFF positive individuals, ranged from 3·4% (95% CI: 0·41-11·7) to 19·8% (95% CI: 13·0-28·3), and from 0% to 2·9% (95% CI: 0·6-8·1) for *A. ceylanicum* (Figure 2). The age distribution of individuals according to their infection status for any hookworm is shown in Figure 3A.

CRs, and AM and GM ERRs for all hookworms (*N. americanus, A. ceylanicum* and *A. duodenale*) by microscopy and mqPCR are shown in Table 4. A large difference in the CR was detected by microscopy (81·5%) and mqPCR (46·4%), whereas the difference between GM ERR was less pronounced for the two methods (Table 4). CRs were similar for *N. americanus* (48·3%) and *A. ceylanicum* (52·6%) whereas GM ERR was higher for the latter parasite (84·5% vs 98·7%).

**Table 4:** Cure rates and egg reduction rates for total hookworms, *Necator americanus, Ancylostoma ceylanicum* and *Ancylostoma duodenale* by microscopy and qPCR.

	Hookworms (n=151)	*N. americanus* (n=143)	*A. ceylanicum* (n=19)	*A. duodenale* (n=1)
Microscopy CR (%)	81·5% (95% CI: 74·3-87·3)	n/a	n/a	n/a
ERR Microscopy GM ERR Microscopy AM	95·8% (95% CI: 92·8-98·3) 40·8% (95% CI: -21·5-84·6)	n/a	n/a	n/a
qPCR CR (%)	46·4% (95% CI: 38·2-54·6)	48·3% (95% CI: 39·8-56·8)	52·6% (95% CI: 28·9-75·6)	0%
qPCR GM ERR qPCR AM ERR	83·2 (95% CI: 77·1-88·1) 72·5% (95% CI: 57·7-83·1)	84·5% (95% CI: 78·6-89·8) 77·6% (95% CI: 63·2-87·2)	98·7% (95% CI: 96·9-99·8) 62·9% (95% CI: 23·4-91·5)	n/a

n/a not applicable

### Predictors for probability of being cured after albendazole administration

**Figure 4:**
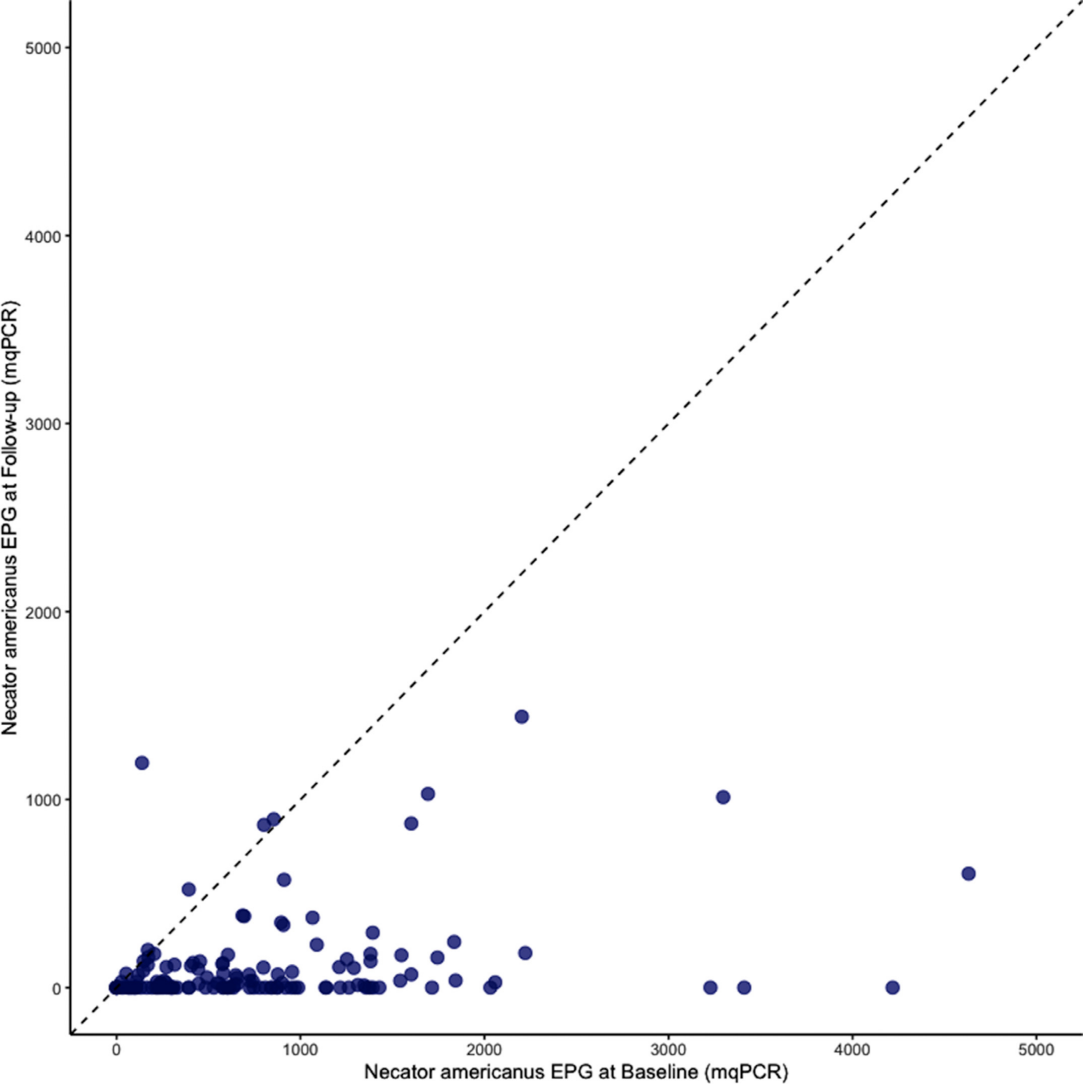
Scatterplot of eggs per gram (EPG) inferred via mqPCR for hookworms at the baseline and after a single treatment with albendazole.

Cure rates for *N. americanus* and *A. ceylanicum* determined using mqPCR (stratified for sex, age groups and single vs. mixed infections) are shown in Table 5. No statistically significant difference was found between sex, age group and single vs mixed infection, with the probability of being cured using the Pearson's Chi-squared test or the Fisher's Exact Test if cell counts were low. The age distribution of people based on the cured outcome (i.e. cured versus not cured) from any of the three hookworm species is displayed in Figure 3B. Further, the impacts of baseline prevalence and baseline EPG (SFF and mqPCR converted values) on cure rates for *N. americanus* and *A. ceylanicum* according to age, sex and baseline EPG values (the sum of *N. americanus* and *A. ceylanicum* EPG) were not found to be significant at the 5% level. A scatterplot of EPG obtained by mqPCR for hookworms at the baseline and after treatment with albendazole are shown in Figure 4. There was strong evidence to reject the null hypothesis “that baseline EPG (microscopy and mqPCR values) has no association with follow-up ERR”. Age, sex and community membership were not found to be statistically significant.

**Table 5:** qPCR-based cure rates for *Necator americanus* and *Ancylostoma ceylanicum* stratified for sex, age groups and single or mixed infections.

	*N. americanus*	*A. ceylanicum*						
	N	Cure rates (%) (95% CI)	X^2^	P-value	N	Cure rates (%) (95% CI)		P-value (Fisher's Exact Test)
Overall	143				19			
Sex								
Male	87	50·6 (39·6-61·5)	0·272	0·602	5	60 (14·7-94·7)		1
Female	56	44·6 (31·3-58·5)			14	50·0 (23-77)		
Age								
6–11	30	53·3 (34·3-71·7)	3·566	0·312	3	66·7 (9·4-99·2)		0·657
12–17	21	52·4 (29·8-74·3)			2	0 (0-84·1)		
18–64	74	50·0 (38·1-61·9)			12	58·3 (27·7-84·8)		
>65	18	27·8 (9·7- 53·5)			2	50·0 (1·3-98·7)		
Infection								
Single	131	49·6 (40·8-58·5)	0·606	0·436	8	37·5 (8·5-75·5)		0·370
Mixed	12	33·3 (9·9-65·1)			11	63·6 (30·9-89·1)

**Figure 5:**
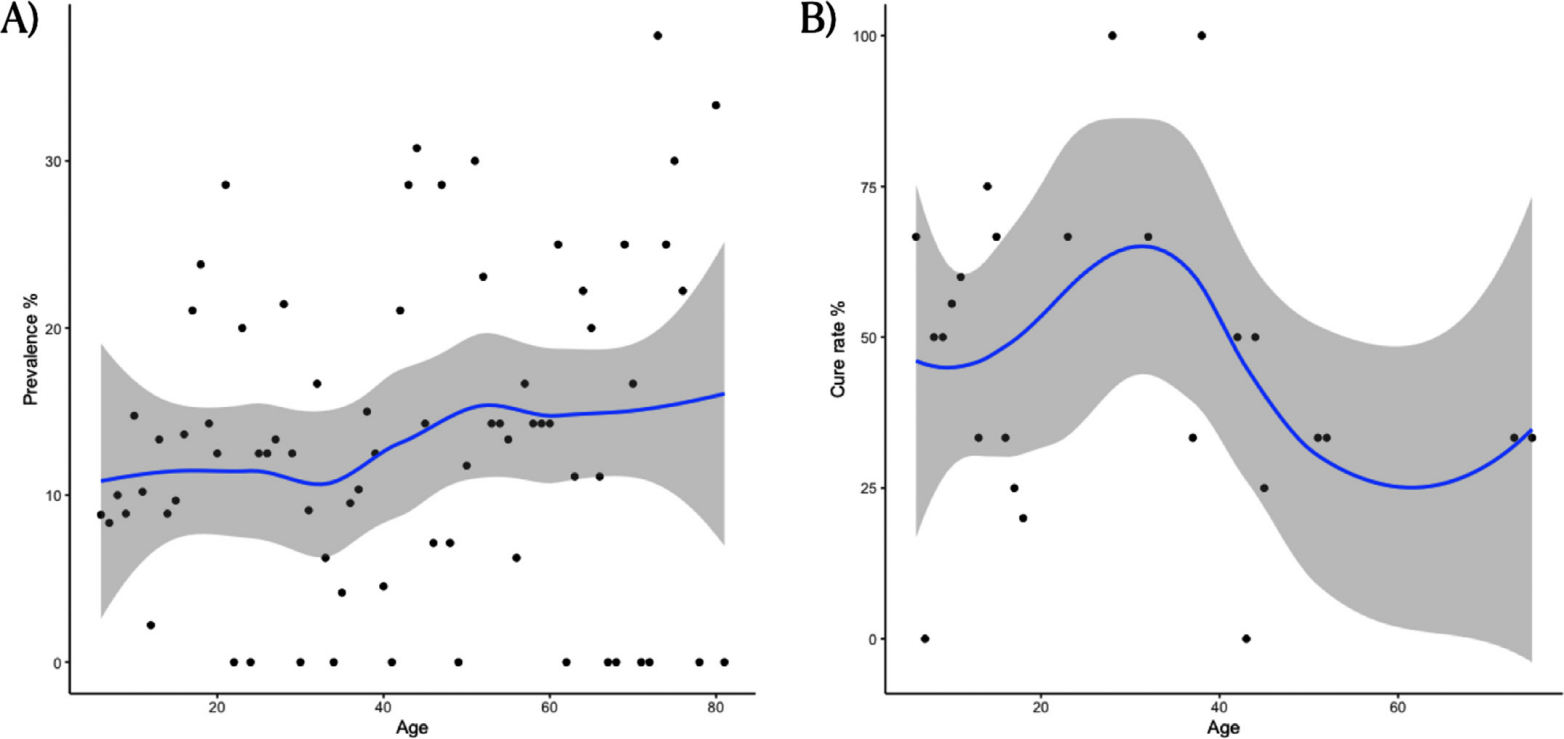
Smoothed age-prevalence at baseline and cure rate-age curves.

There was evidence of an association between the fixed effects of age and boiling drinking water during the wet season on the odds of being cured, holding all other variables constant (Table 3). We estimated that as age increases by one year the odds of being cured decreases by between 0·35% and 3·71% on average, holding boiling water in the wet season constant, while adjusting for the community effect (95% CI). We estimated the odds ratio of being cured for boiling drinking water in the wet season to be between 1·02 and 6·82 (95% CI), holding age constant, while adjusting for the community effect. Smoothed age-prevalence at baseline and cure rate-age curves are shown in Figure 5.

## Discussion

Here, we assessed (i) individual participant risk factors associated with hookworm infection, (ii) CRs and ERRs of albendazole against *N. americanus* and *A. ceylanicum*, and (iii) factors that influence the success of chemotherapeutic interventions employing distinct diagnostic techniques and analytical approaches. This study provides evidence that the outcomes of risk factors for hookworm infections and for the efficacy of treatment against hookworm species are highly dependent on the statistical approach and diagnostic methods used.

By the mean of a generalized linear mixed model, we identified a higher tendency of older individuals to be infected by hookworms than younger members of the communities, with the odds of being infected increasing at a faster rate for females than for males as age increases. Conversely, no difference was found between infection at the baseline and age classes using a Pearson's Chi-squared test, highlighting how categorising age classes may present an overly simplistic picture that could obfuscate the evaluation of a long-term impact of control strategies on community transmission.

More than 80% of the infected individuals were above the ‘age-target’ of the current WHO control guidelines (2-4 years, pre-SAC and 5-12 years, SAC) – this large population of untreated people serves as reservoir for hookworm infections, guaranteeing the perpetuation of community-wide transmission, thus hindering the success of targeted-PC.

The disproportionate number of people infected by hookworms may be the result of a negative synergistic effect of currently-adopted control strategies for soil-transmitted helminths (STHs) and the intrinsic infection dynamic that each parasitic species deploys to infect their host(s). For instance, *A. lumbricoides* and *T. trichuira* are transmitted through the ingestion of eggs, an event that more likely occurs in children than in adults, while hookworm infections are mainly acquired through skin penetration of infective larvae from the environment, indiscriminately affecting people of all ages. ^2^^,^^33^ In addition, a protective immunity following repeated exposures to hookworms does not seem to develop, leaving individuals of all age groups susceptible to infection. ^34^

This latter aspect has a clear impact on the age distribution of people infected by different species of STHs, with the proportion of hookworm-infected people steadily rising from childhood to late adulthood, and ascariasis and trichuriasis declining with age. The effect of these age-related practices has repercussions for the success of chemotherapeutic interventions, with current STHs elimination programs likely to have a greater impact on ascariasis and trichuriasis, while having little or no effect on the community-wide transmission of hookworms. ^35^ Therefore, high PC coverage of adults has been deemed to be more important for hookworms than for ascariasis transmission due to different age-patterns in infection levels. ^13^^,^[35], [36], [37] Similarly, we estimate that, as age increases by one year, the odds of being cured decreases by between 0·35% and 3·7% on average, with younger individuals more likely to clear an infection than the older population (95% CI). Targeted drug administration would leave the elderly and people in their working age untreated against hookworms – which are known to promote and thriving in poverty-stricken communities.^38^ Shifting from a targeted to a mass drug administration can significantly reduce hookworm prevalence in all age classes, and decrease the risk of reinfection of pre-SAC, SAC^39^ and WCBA and, thus reduce morbidity in the latter high risk groups. A sustained decline in the number of infected people at the community level will enable the reduction of poverty and increase socioeconomic development, and will save millions of people, with already naturally low iron reserves, from the negative impact of iron-deficiency anaemia. In addition, we found evidence that boiling drinking water influences the probability of both being infected and cured by hookworms, as a likely effect of increased infection awareness and/or better living conditions, emphasising the importance of water, sanitation and hygiene in relation to STHs control.^40^

We detected hookworm infection in 13·5% of 1,232 people enrolled in this study by SFF. However, this figure might be an underestimation of the true prevalence of infection, given the lower sensitivity of microscopic methods compared to mqPCR. In addition, enrolling individuals that had to voluntarily present themselves to the pagoda might have attracted people that were more compliant with hygiene and deworming practices, representing a potential sampling bias. While this might have had an influence on the percentage of people infected by hookworms in the cross-sectional survey, this sampling procedure has little or no effect on the efficacy rates of albendazole herein reported. Moreover, we did not detect any effect of previous deworming on the likelihood of being infected and cured.

In the present study, we used a molecular approach to characterise species of hookworms infecting people who live in resource-poor communities in Cambodia, and to calculate infection intensity at baseline and follow-up, being instrumental for the evaluation of species-specific CR and ERR following albendazole administration. Given that conventional coproscopic techniques have low sensitivity for the detection of low-intensity hookworm infections^25^ molecular techniques are crucial for the diagnose of such infections. This is the case of people living in communities that have received multiple treatments within MDA, during post-MDA surveillance, or when large worm burden in the host results in a decreased production of eggs. [41], [42], [43] Therefore, qPCR should be used instead of conventional coproscopic techniques to better estimate the distribution and intensity of STH infections in endemic areas and to monitor the effectiveness of deworming programs. This holds true for hookworms, but not for other STHs, for the tendency of their eggs to collapse and disappear when using conventional techniques, such as the Kato Katz thick smear. ^43^^,^^44^ The use of microscopy-based methods which have low sensitivities leads to an underestimation of prevalence of infection and an overestimation of efficacy of treatment programs, because individuals excreting low numbers of eggs are misclassified as cured.

In the present study, a clear reflection of this is the large difference in the CR of albendazole against hookworm infections when conventional (81·5%) vs molecular (46·4%) methods were used. While the CR for microscopy obtained was similar to that reported in other studies, the number of people who were test-positive (shedding eggs) by molecular means following albendazole administration was significantly higher than reported in systematic reviews and meta-analyses of efficacy studies conducted using conventional coproscopic methods. ^24^^,^^45^ Conversely, ERR was similar to that previously reported using either microscopic^24^^,^^45^ or molecular methods.[41]

We found no evidence that baseline prevalence and baseline EPG (SFF and mqPCR) had impacts on CR and ERR for each species of hookworm, however future studies involving a larger sample of hookworm positive individuals at baseline, may aid in addressing this research question. We did observe a difference in AM and GM ERR, as estimated based on results for SFF and mqPCR, likely related to an effect of few people shedding a high number of eggs after albendazole administration. Whether this relates to a lack of susceptibility or benzimidazole resistance^46^ remains to be established. Low CRs have also been recorded previously for *T. trichiura* in areas with a long history of PC^47^ and for hookworms in Lao PDR^27^ after albendazole administration. The possible aetiologies of these treatment failures warrant investigation.

The availability of reliable diagnostic techniques becomes central when aiming for the WHO 2030 morbidity control target, ^48^ when moving towards a breakpoint of transmission, and when monitoring for “bounce-back” effects or the re-emergence of neglected hookworms. ^49^ Here, with the aid of a molecular approach, we report the first efficacy data against *A. ceylanicum* infection in humans. Until recently, human hookworm infections were only attributed to *N. americanus* and *A. duodenale*, due to an inability to unequivocally distinguish eggs of all known hookworm species by microscopy. However, with the advent of molecular techniques, we are now observing a steep rise in the number of countries identifying *A. ceylanicum* as agent of hookworm infections in humans, with this zoonosis now considered the second most common hookworm in Southeast Asia and the Western Pacific.^15^^,^ ^50^ Recent work shows that the morbidity of *A. ceylanicum* is linked to a platelet inhibitor in adult parasites involved in the pathogenesis of hookworm-associated intestinal haemorrhage and iron deficiency anaemia.^51^ However, to date, no data have been available on the efficacy of anthelminthic drugs against *A. ceylanicum*, despite an estimated 100 million people estimated to be infected with this parasite.^14^ Similarly, despite report of infections in humans showing clinical signs such as eosinophilia, diarrhoea, melena, dyspnoea, and anaemia, studies elucidating *A. ceylanicum*-associated morbidity at a population scale have not yet been performed.[14], [15], [50]

A single dose of albendazole showed promising efficacy against *A. ceylanicum*, with comparable CRs and ERRs to other human-only hookworm species. However, *A. ceylanicum* has dogs as the key reservoir of infection, which emphasises the importance of implementing One Health intervention programs in areas where this zoonotic hookworm occurs at high prevalence in both human and canine populations.^14^^,^^50^ The increasing success of current human-oriented STHs control strategies may mask the relevance of animal reservoirs and could lead to a resurgence of *A. ceylanicum* infection, and seriously impact the health of people living in poor socio-economic settings. ^14^ Although STH species are treated collectively with regard to control strategies, refined guidelines are needed for a sustained reduction in morbidity based on local epidemiological knowledge of the dominant species, their transmission patterns, and their prevalence and infection intensity.

To date, notwithstanding the great efforts and sustained reduced morbidity achieved worldwide through targeted treatment programs, hookworms still cause chronic human infections associated with malnutrition, growth stunting and reduced cognitive abilities and intellectual capacity. The findings presented in this study show that the adoption of refined diagnostic techniques is central to monitoring hookworm infection(s) and the success of control strategies, which can ultimately aid in reducing associated morbidity in human populations.

The approach taken is likely to be directly applicable to other parts of Southeast Asia and the Western Pacific, where specific epidemiological conditions might hamper the success of targeted treatment programs and provides researchers and policy makers with crucial information to underpin the development of refined strategies to control hookworm infections.

## Contributors

**Vito Colella**: investigation, literature search, visualization, methodology, data analysis, data interpretation, writing – original draft, and writing– review & editing.

**Virak Khieu**: investigation, methodology, data interpretation, project administration, validation, and writing– review & editing.

**Andrew Worsley**: formal analysis, methodology, software, validation, visualisation, and writing– review & editing.

**Dammika Senevirathna**: formal analysis and methodology.

**Sinuon Muth**: formal analysis, methodology, and writing– review & editing.

**Rekol Huy**: formal analysis, methodology, and writing– review & editing.

**Peter Odermatt**: data interpratation, methodology, validation, and writing– review & editing.

**Rebecca J. Traub**: conceptualisation, data curation, funding acquisition, investigation, project administration, supervision, validation, and writing– review & editing.

## Data sharing statement

All relevant data from this study are contained in the article. All other data are available from the corresponding author upon reasonable request following the publication of this article.

## Declaration of Competing Interest

The authors declare no conflicts of interest.

## Appendix Supplementary materials

Image, application 1
